# F-Box Gene D5RF Is Regulated by Agrobacterium Virulence Protein VirD5 and Essential for Agrobacterium-Mediated Plant Transformation

**DOI:** 10.3390/ijms21186731

**Published:** 2020-09-14

**Authors:** Shaojuan Zhang, Zhuo Chen, Fei Huang, Yafei Wang, Meizhong Luo

**Affiliations:** College of Life Science and Technology, Huazhong Agricultural University, Wuhan 430070, China; zhangsj@webmail.hzau.edu.cn (S.Z.); chenzhuo@webmail.hzau.edu.cn (Z.C.); feihuang@sjtu.edu.cn (F.H.); wangyafei@webmail.hzau.edu.cn (Y.W.)

**Keywords:** *Agrobacterium*-mediated transformation, VirD5, D5RF (VirD5 response F-box protein)

## Abstract

We previously reported that the *Agrobacterium* virulence protein VirD5 possesses transcriptional activation activity, binds to a specific DNA element D5RE, and is required for *Agrobacterium*-mediated stable transformation, but not for transient transformation. However, direct evidence for a role of VirD5 in plant transcriptional regulation has been lacking. In this study, we found that the Arabidopsis gene *D5RF* (coding for VirD5 response F-box protein, *At3G49480*) is regulated by VirD5. *D5RF* has two alternative transcripts of 930 bp and 1594 bp that encode F-box proteins of 309 and 449 amino acids, designated as D5RF.1 and D5RF.2, respectively. D5RF.2 has a N-terminal extension of 140 amino acids compared to D5RF.1, and both of them are located in the plant cell nucleus. The promoter of the *D5RF.1* contains two D5RE elements and can be activated by VirD5. The expression of D5RF is downregulated when the host plant is infected with *virD5* deleted *Agrobacterium*. Similar to VirD5, D5RF also affects the stable but not transient transformation efficiency of *Agrobacterium*. Some pathogen-responsive genes are downregulated in the *d5rf* mutant. In conclusion, this study further confirmed *Agrobacterium* VirD5 as the plant transcription activator and identified *Arabidopsis thaliana* *D5RF.1* as the first target gene of VirD5 in regulation.

## 1. Introduction

*Agrobacterium* (*Agrobacterium tumefaciens*) is a soil-borne pathogen that transforms plant cells into tumor cells by the delivery of an oncogenic piece of DNA from its Ti (tumor-inducing) plasmid [1,2]. Therefore, the Ti plasmid via *Agrobacterium* is used as an important gene vector for the genetic modification of not only a wide range of dicotyledonous plants and certain monocotyledonous plants [3,4], but also fungi [5]. During the *Agrobacterium* infection process, a single-stranded copy of a segment of the Ti plasmid, called the “T-DNA strand”, is transferred into host cells [3,6]. The process is mediated by at least five virulence effector proteins (VirE2, VirE3, VirF, VirD2, and VirD5) [7]. VirD2 is covalently attached to the 5′ end of the T-DNA strand to form the VirD2–T-strand. The VirD2–T-strand and other virulence effector proteins are translocated into plant cells via the type IV secretion system (T4SS) of the bacterium that is composed of VirB1-11 and VirD4 proteins [8,9,10,11]. Within the host cells, VirE2 and VirD2–T-strand are assembled as the VirD2–T-strand–VirE2 nucleoprotein complex (T-complex), in which numerous VirE2 proteins coat the T-DNA to protect the T-DNA from degradation [10,11,12]. VirE2 also interacts with the host protein AtVIP1 (VirE2-interacting protein 1), a basic leucine zipper (bZIP)-family transcriptional factor, which may promote transfer of the T-complex to the nucleus and serve as part of the defense response [13]. However, it was reported that a *vip1* mutant was transformed equally well as the wild type, so the importance of VIP1 in transformation was questioned [14]. There are two major models for the mechanism of T-DNA integration into the plant genome: the strand-invasion model and the double-strand break repair and integration model. The former model assumes that the single-strand VirD2-T-strands search and utilize the microhomologous regions between the T-DNA border sequences and the plant genome sequences, and then the T-strands locally invade and melt into the host DNA of the target sites [6,15,16]. The latter model assumed that the single-strand VirD2-T-strands were first replicated as double-strands in the plant nucleus, and then integrated into the double-strand breaks in the host genome [6,17,18]. It has been shown that T-DNA integration relies on PolQ in *Arabidopsis thaliana* [19]. Studies have shown that several plant genes are also important for T-DNA integration into plant DNA including those encoding histone H2A [20], histone H3 [21], the histone H3 chaperone SGA1 [22], the histone deacetylases HDT1 and HDT2, and the NOT domain transcriptional factor VIP2 (VirE2-interacting protein 2) [23]. VIP2 regulates the transcription levels of genes coding for the histones and histone deacetylases. In *vip2* mutants, genes coding for the histones and histone deacetylases were repressed, and a *vip2* mutant was completely sensitive to transient transformation, while *vip2* mutant and VIGS-silenced plants were less sensitive to stable transformation [23].

Before the T-strand integrates into plant chromatin, the coat proteins need to be removed. Two proteins, VirF [24] and VBF (AtVIP1-Binding F-box protein), are considered to be involved in the T-complex uncoating process [25]. VirF protein is a host range factor, which is present in the octopine Ti plasmid [26,27], but not in the nopaline-type and agropine-type Ti plasmids. It contains an F-box structure and thus may be incorporated into a Skp1-Cdc53-F-box (SCF) ubiquitin-ligase (E3) complex in the host cells [28] to degrade VirE2, AtVIP1, and some other host proteins in the nucleus by directly binding to them [24,29]. The VBF protein is an endogenous F-box protein identified in some plants such as *Arabidopsis thaliana*, and in those plants, VirF is required for tumorigenicity. Its expression is induced by *Agrobacterium*. It may take over from VirF [25]. Furthermore, the *A. tumefaciens* virulence effector protein VirE3 may also play an important role in coating-uncoating T-DNA. VirE3 interacts with pBrp (a TFIIB-like transcriptional factor) and stimulates transcription of host genes including VBF [30,31]. Recently, it has been reported that VirE3 can be used as an anchor protein of VirE2 and helps VirE2 in aggregating at the host entry site to protect T-DNA [32].

VirD5 is another *A. tumefaciens* virulence effector protein and conserved in all strains of *A. tumefaciens* and *A. rhizogenes*. It consists of 833 amino acids and contains three putative nuclear localization signals (NLSs), and putative helix–turn–helix and helix–loop–helix domains [12,33]. Induced expression of VirD5 in Arabidopsis and yeast inhibits growth and gradually leads to death [34]. VirD5 shows localization at the kinetochores in the nucleus of the yeast cells and interacts with the host Spt4 protein. Without the Spt4, VirD5 localization was translocated and its toxicity was strongly reduced. It is a prokaryotic virulence protein that interferes with mitosis by promoting chromosomal instability [34]. Recently, it was shown that VirD5 interacts with the conserved mitotic Aurora kinase Ipl1, resulting in the detachment between the kinetochore and spindle microtubule by a phosphorylating substrate in vivo, and stimulating its activity [35]. VirD5 is also considered to stabilize VirF in host cells by mutual interaction [36].

Our group has been working on the mechanism of *Agrobacterium*-mediated plant genetic transformation and mainly focused its attention on *Agrobacterium* VirD5 elicited plant responses [33,37]. We found that VirD5 has different functions. It interacts with Arabidopsis VIP1, forms ternary complex with AtVIP1 and VirE2 in the plant cell nucleus, and competes with VBF for binding to AtVIP1 to prevent VirE2 and AtVIP1 from being rapidly degraded via UPS (ubiquitin proteasome system). It also has transcriptional activation activity in yeast, forms homodimers in vivo and in vitro, and binds to a specific DNA element (D5RE, CCGCNC/GNGCGG). Yeast one-hybrid experiment confirmed its function as a transcriptional activator [33]. We further found that VirD5 is required for plant stable transformation, but not for transient transformation, and *virD5* deletion *A. tumefaciens* mutant strains affected the tumorigenesis efficiency on the stems of *tomato* and the root segments of Arabidopsis. It competes with the host protein CBPs (cap-binding proteins, CBP20 and CBP80) for binding to host protein AtVIP2 [37]. Recently, we identified a VIP1 homologue in rice [38], indicating that rice may have a similar mechanism for *Agrobacterium*-mediated transformation.

However, up to date, direct evidence for a role of VirD5 in transcriptional regulation in plants has been lacking. Here, we report a plant gene that is regulated by VirD5. Its expression is downregulated upon deletion of *virD5*. It encodes a F-box protein with RNI-like/FBD-like domains and is located in the plant cell nucleus. We named this protein “D5RF” (VirD5 response F-box protein). Genetic evidence strongly indicated that D5RF is necessary for *Agrobacterium*-mediated transformation.

## 2. Results

### 2.1. The Arabidopsis D5RF Gene Is Regulated by VirD5 at the Transcription Level

By searching the 500 bp sequence region upstream of ATG of the Arabidopsis database, we found a large number of genes in the Arabidopsis genome that contain the D5RE element. In order to select more believable candidates to determine the host target genes regulated by VirD5, we set two screening conditions: (1) the 500 bp promoter region (on both +/− strands) contains at least two D5RE elements (as VirD5 can form dimers and the yeast one hybrid experiment also showed that there may be two continuous DNA binding domains on VirD5 [33]); (2) The annotated function of the candidate gene is involved in the host plant disease resistance/sensibility or the process of *Agrobacterium* infection. Using these criteria, we identified 13 candidate Arabidopsis genes. In this paper, we report the results for six genes (Supplementary Table S1).

**Figure 1:**
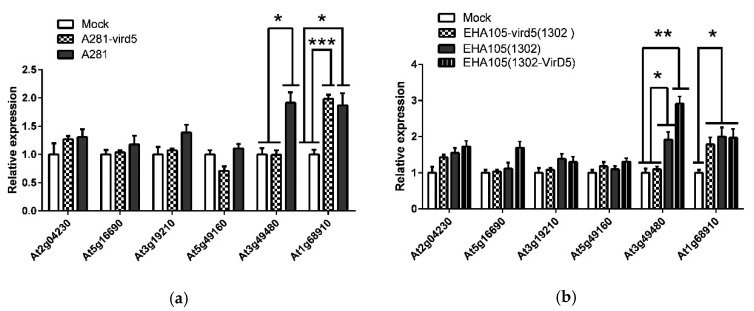
The expression of the genes possibly directly regulated by VirD5. (**a**) The expression in Arabidopsis leaves syringe (needleless) infiltrated with *Agrobacterium* A281 or its *virD5* deletion mutant A281–*vird5*. (**b**) The expression in Arabidopsis leaves syringe (needleless) infiltrated with *A. tumefaciens* EHA105 and its *virD5* deletion mutant EHA105-*vird5*, either carrying a binary vector pCAMBIA1302 or pCAMBIA1302:VirD5. Leaf samples from the infiltrated area were collected at 48 h after inoculation, and total RNA was isolated for QRT-PCR. RNA from the Mock (10 mm MgCl_2_ solution) infiltrated Arabidopsis leaves collected at 48 hpi was used as a calibrator to determine the relative number of gene transcripts. All data are the means ± SE from three biological replicates. The data were made into graphs using GraphPad PRISM 5 software (La Jolla, CA, USA). *, *p* < 0.1; **, *p* < 0.01; ***, *p* < 0.001.

In order to verify if these genes are truly regulated by VirD5, we performed expression analyses by real-time quantitative PCR (QRT-PCR) on Arabidopsis leaves treated with the two pairs of *Agrobacterium* strains: the tumorigenic strain A281 and the disarmed EHA105 (both are succinamopine-type strains) and their *virD5* deletion mutants A281-*vird5* and EHA105-*vird5*. In the first experiment, Arabidopsis leaves were separately syringe (needleless) infiltrated with A281 and its *virD5* deletion mutant (A281-*vird5*). The buffer (10 mm MgCl_2_ solution, Mock) was used as the control. We sampled leaves at 48 hpi (hour postinfection) and determined the expressions of the putative genes by QRT-PCR. With infection of A281, the expression of the *At3g49480* gene was increased approximately two-fold. However, with infection of the strain A281-*vird5* or in the Mock treatment with the buffer, the expression of *At3g49480* was not significantly altered. This result indicates that the expression of the *At3g49480* gene is positively regulated by *Agrobacterium* VirD5. Although the expression of the *WIT2* (*At1g68910*) gene was also increased approximately two-fold with infection of A281, it was increased to a similar extent with infection of A281-*vird5*, indicating that the expression of the *WIT2* gene was not regulated by VirD5, but probably regulated by other *Agrobacterium* virulence effector proteins. The expressions of other genes were not significantly changed (Figure 1a). In the second experiment, individual leaves of Arabidopsis plants were syringe (needleless) infiltrated with EHA105 and its *virD5* deletion mutant EHA105–*vird5*, either carrying a binary vector pCAMBIA1302 or pCAMBIA1302:VirD5. Leaf samples from the infiltrated area were collected at 48 h after inoculation, and total RNA was isolated for QRT-PCR. RNA from the buffer (Mock) infiltrated Arabidopsis leaves collected at 48 hpi was used as a calibrator to determine the relative number of gene transcripts. As in the first experiment, the expressions of *At3g49480* in EHA105 (carrying pCAMBIA1302 or pCAMBIA1302:VirD5) infiltrated Arabidopsis leaves were increased significantly compared with those in EHA105–*vird5* (carrying pCAMBIA1302) infiltrated or buffer treated Arabidopsis leaves. Supplementing VirD5 (pCAMBIA1302:VirD5) further increased the expression of *At3g49480*. All EHA105 infiltrated samples showed an increased expression of the *WIT2* (*At1g68910*) gene, but deleting and supplementing VirD5 did not affect the expression of the *WIT2* (*At1g68910*) gene (Figure 1b). This experiment further demonstrated that VirD5 positively regulates the expression of the *At3g49480* gene but not that of the *WIT2* (*At1g68910*) gene. Again, the expressions of other genes were not significantly changed. Therefore, we focused our in-depth research on the *At3g49480* gene in this study and named the protein as “D5RF” (VirD5 response F-box protein).

### 2.2. Bioinformatics Analysis and Subcellular Localization of D5RF

**Figure 2:**
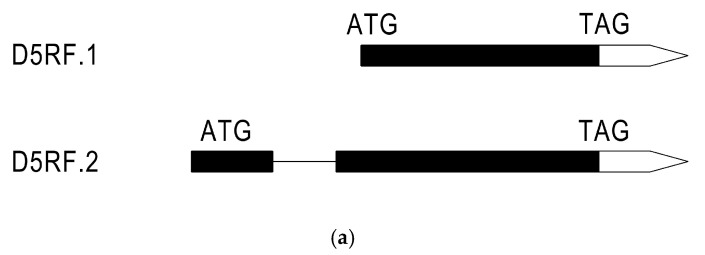

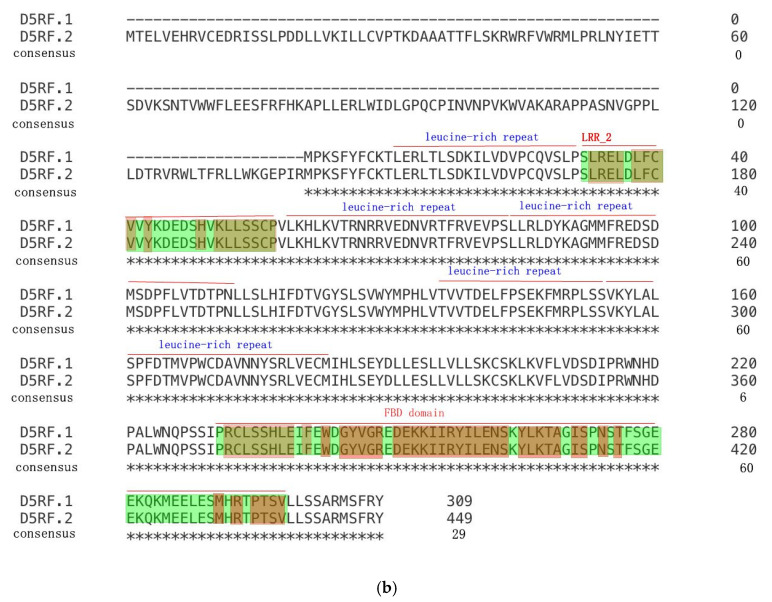
Gene structure and amino acid sequence alignments of the two D5RF open reading frames. (**a**) Schematic representation of the *D5RF* genomic organization with exons (black boxes) and introns (lines between exons). White boxes indicate the 3′-UTRs. (**b**) Amino acid sequence alignments of the two D5RF. Conservative domains are indicated in green, and important sites are indicated in gold.

By applying a general bioinformatics analysis, we found that *D5RF* has two alternative transcripts of 930 bp and 1594 bp (GenBank Accessions NM_114808 and NM_001339422.1), which encode F-box proteins of 309 and 449 amino acids with unknown functions, designated as D5RF.1 and D5RF.2, respectively. D5RF.2 has a N-terminal extension of 140 amino acids compared to D5RF.1 (Figure 2a,b). Two D5RE elements were located in the promotor of the *D5RF.1* (Supplementary Figure S1). However, no D5RE elements were found in the 2000 bp sequence region upstream of the translational start site of *D5RF.2*.

**Figure 3:**
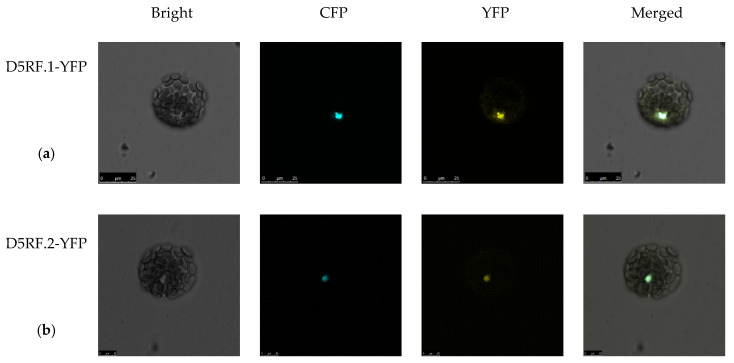
Subcellular localization assay for D5RF.1 and D5RF.2 proteins. The D5RF.1-YFP or D5RF.2-YFP vectors were separately co-transferred with the 35S::CFP-Ghd7 vector into Arabidopsis protoplast cells (**a**,**b**). The 35S::CFP-Ghd7 was used as the nucleic marker. The confocal image was observed using a confocal laser scanning microscope.

The subcellular localization of a protein is an important clue in understanding its function. Both D5RF.1 and D5RF.2 were predicted to be localized in the nuclear by BaCelLo (http://gpcr2.biocomp.unibo.it/bacello/pred.htm) [39], while in the cytoplasm by WoLF RSORT, the two online programs that are used to predict the subcellular localization of plant proteins. To detect whether the D5RF protein can be transported into the plant cell nucleus, we performed a subcellular localization assay. The transient expression constructs 35S::D5RF.1–YFP or 35S::D5RF.2–YFP were separately co-transferred into Arabidopsis protoplast cells with the construct 35S::Ghd7-CFP. The Ghd7 was used as a cell nucleus localization marker [40,41]. The results showed that the YFP signals of both 35S::D5RF.1–YFP and 35S::D5RF.2–YFP were mainly observed in the nucleus (Figure 3a,b).

### 2.3. VirD5 Can Activate the Promotor of D5RF.1

**Figure 4:**
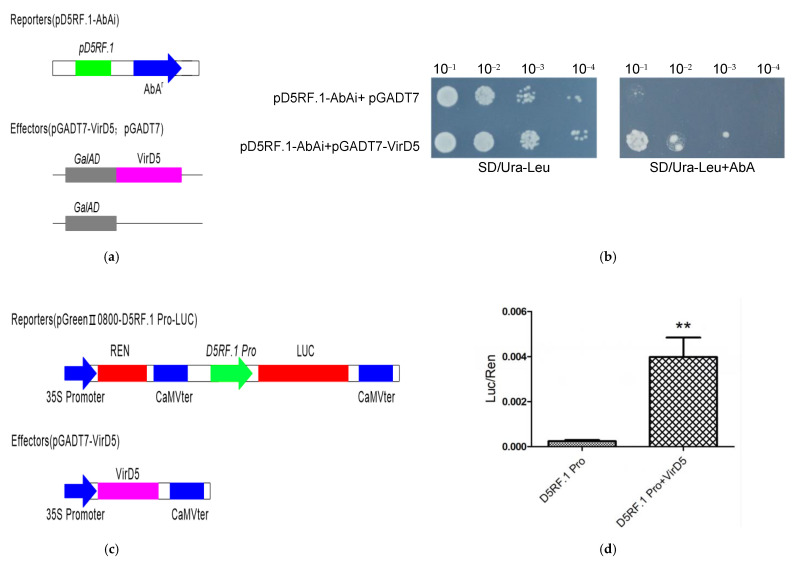
Verification of the VirD5 activity on the *D5RF.1* promoter. (**a**) Schematic representation of the constructs used for yeast one-hybrid assay. (**b**) Yeast one-hybrid assays. The PGADT7:VirD5 and pAbAi reporter under the control of pD5RF.1 were co-transferred into yeast strain Y1H Gold. The transformed yeast cells were first cultured on SD minus Uracil and Leucine (SD/-Ura-Leu) medium, then the yeast colonies were resuspended in sterile ddH_2_O, adjusted to an OD_600_ of 0.8, and cultured on SD/-Leu-Ura minimal medium and SD/-Leu-Ura minimal medium with 500 ng.mL^−1^ Aureobasidin A. (**c**) Schematic representation of the constructs used for dual-luciferase assays. The reporter construct contains the firefly luciferase gene (LUC) driven by *D5RF.1* promoter, and the Renilla luciferase gene (REN) driven by the CaMV 35S promoter. The effector construct contains *virD5* driven by the CaMV35S promoter. (**d**) Dualluciferase assays. The reporters and effectors were co-expressed in *N. benthamiana*, and both REN and LUC activity were measured. The relative LUC activities normalized to the REN activities are shown (LUC/REN). Data represent mean ± SE of two biological replicates. The statistical significances were determined using the t-test. ** *p* < 0.01.

To test whether VirD5 can bind to the promoter of Arabidopsis *D5RF.1*, we performed one-hybrid assays in yeast and dual-luciferase assays in *Nicotiana benthamiana*. For yeast one-hybrid assays, the 500 bp sequence upstream of the translational start site of *D5RF.1* was constructed in the pAbAi vector and the *virD5* sequence was constructed in the pGADT7 vector (Figure 4a). As shown in Figure 4b, when the constructs pD5RF.1-AbAi and pGADT7-VirD5 were co-transferred into the yeast cells, the yeast cells could grow on the SD/Ura-Leu+AbA medium, indicating that VirD5 can bind to the Arabidopsis *D5RF.1* promoter to activate the expression of the reporter gene. For dual-luciferase assays, the reporter vector was constructed in which the firefly luciferase (LUC) gene was driven by the *D5RF.1* promoter and the renilla luciferase (REN) gene was driven by the CaMV 35S promoter and the effector vector was constructed in which the *virD5* was driven by the CaMV 35S promoter (Figure 4c). As shown in Figure 4d, in the absence of VirD5, the LUC to REN ratio was low, while in the presence of VirD5, the LUC to REN ratio was significantly increased. These data further demonstrated that VirD5 can bind to the *D5RF.1* promoter and activate the firefly luciferase gene expression.

### 2.4. D5RF Is Required for Plant Stable Transformation, But Not for Transient Transformation

We checked whether D5RF was involved in the *Agrobacterium* infection process. First, we identified an Arabidopsis T-DNA mutant *d5rf* from the SALK Arabidopsis Stock Center. To isolate the *d5rf* mutant, we searched the TAIR (The Arabidopsis Information Resource) flanking sequence database, and found a T-DNA insertion approximately 200 bp (Supplementary Figure S2a) downstream of the translational start site of *D5RF.1*. Using three PCR primers (P1, P2 and P3), we identified that the seeds from the SALK Arabidopsis Stock Center were all homologous insertion *d5rf* mutants (Supplementary Figure S2b). RT-PCR analysis showed that the expression level of *D5RF* was greatly depressed in the T-DNA insertion homozygous plants (Supplementary Figure S2c).

**Figure 5:**
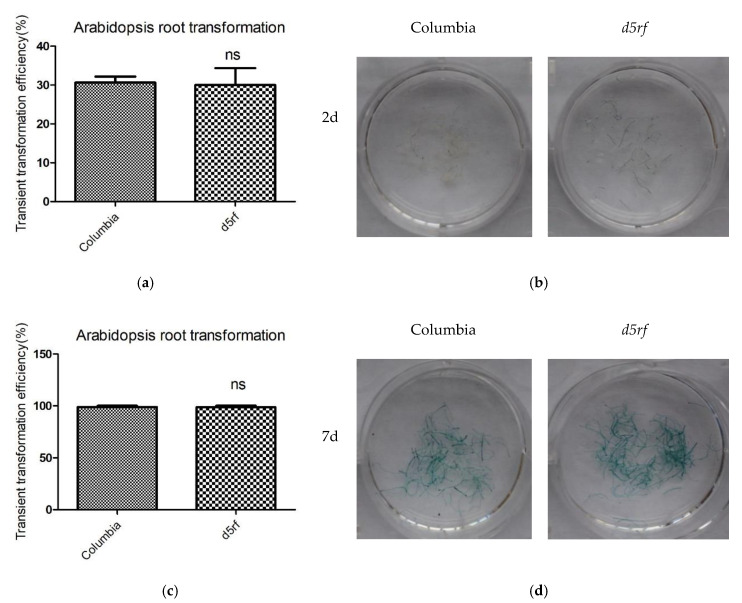
Transient transformation efficiencies of the wild-type and *d5rf* plants. Root segments of the wild-type and *d5rf* plants were inoculated with the *A. tumefaciens* strain EHA105 carrying the *uidA* gene within the T-DNA. Two (**a**,**b**) or seven days (**c**,**d**) later, the inoculated root segments were collected and stained with X-Gluc. The experiment was repeated three times. The transient transformation efficiency was calculated as: no. of root segments stained in blue/no. of root segments infected. All of the data are means ± SD from three biological replicates. The data were made into graphs using GraphPad PRISM 5 software (La Jolla, CA, USA). ns, none significant.

**Figure 6:**
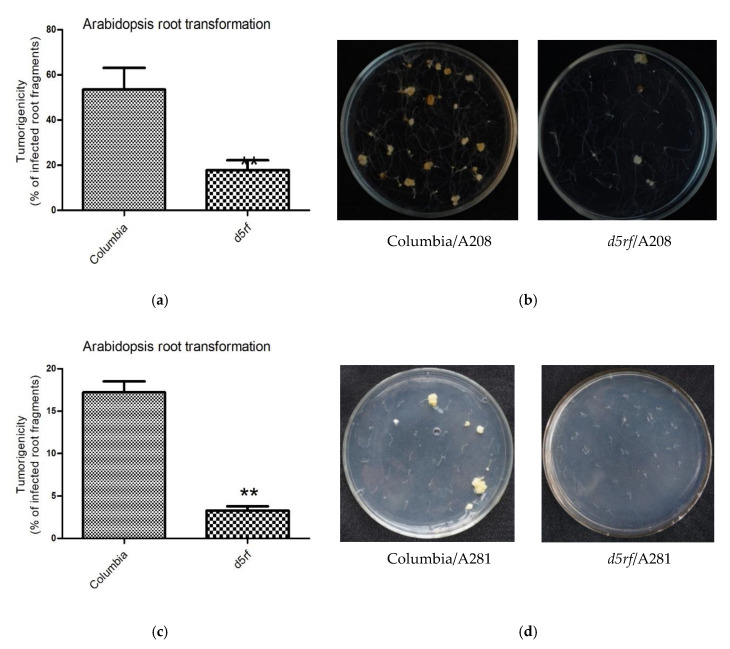
Stable transformation assays. Root segments of wild-type and *d5rf* mutant plants were infected with the tumorigenic strain *A. tumefaciens* A208 (nopaline strain), and tumors incited on the root segments were visualized and scored 3–4 weeks after infection (**a**,**b**). Root segments of wild-type and *d5rf* mutant plants were infected with the tumorigenic strain *A. tumefaciens* A281 (Succinamopine strain), and tumors incited on the roots were visualized and scored 30 d after infection (**c**,**d**). The stable transformation efficiency was calculated as: no. of root segments with tumor/no. of root segments infected. **, *p* < 0.01. Data are means ± SE from five (**a**) or three (**c**) biological replicates.

Then, we conducted transient and stable root transformation assays [42] to determine the role of D5RF in *Agrobacterium*-mediated transformation. In the transient transformation experiments, the *A. tumefaciens* strain EHA105 carrying the *uidA* gene was used to infect Arabidopsis root segments. β-glucuronidase (GUS) analysis showed that no significant difference was observed between the wild type (Columbia; Col-0) and *d5rf* (Figure 5), indicating that D5RF does not function in transient transformation processes. In Arabidopsis stable transformation experiments, either *A. tumefaciens* tumorigenic strain A208 or A281 were used to infect Arabidopsis root segments. The results showed that *d5rf* yielded fewer tumors (decreased for more than threefold) compared with the wild-type plants (Figure 6), indicating that D5RF, as VirD5, plays a role in stable transformation processes.

### 2.5. Pathogen-Responsive Genes Are Possibly Downregulated in d5rf

To gain insights into the biological role of D5RF in plants, we conducted a comprehensive survey of global gene expression employing high throughput-RNA sequencing to quantify the variations in transcript abundance between wild-type Columbia-0 (Col-0) seeding and *d5rf* seeding. Six to seven billion reads were obtained for each library. Most of these reads were mapped to the Arabidopsis reference genome (Supplementary Table S2). Comparative analyses between Col-0 and *d5rf* showed that 2967 unique genes with parameters of |log2FC| > 1 and *p*-value < 0.05 were constitutively differentially expressed. Of these, 1555 genes had a higher transcript abundance and 1412 genes had lower transcript abundance in *d5rf* compared with Col-0 (Supplement Table S3). The regulated genes were grouped into classes using the Gene Ontology (GO) classification adopted by TAIR. Functional classification of the 2967 differentially expressed genes indicated that they are involved in a variety of functions including hormone signaling, defense response, cellular biosynthesis, and nucleic acid metabolism (Supplementary Table S4). Categories such as ‘nucleic acid binding transcription factors’, ‘transcription factor activity’, ‘response to reactive oxygen species sequence−specific DNA binding’, ‘ADP binding’, and ‘peroxidase activity’ are overrepresented among upregulated genes. In contrast, the categories ‘defense response’, ‘photosynthesis’, ‘photosystem’, ‘thylakoid part’, and most of ‘photosystem’ were all overrepresented among downregulated genes (Supplementary Table S5). This implies that the mutation of the *D5RF* gene may not only inhibit the photosynthesis and defense related genes, but also induce the nucleic acid metabolism of plants. The RNA-Seq expression results of the downregulated and some pathogen-responsive related genes were validated using QRT-PCR (Supplementary Figure S3).

## 3. Discussions

The *Agrobacterium* virulence protein VirD5 is one of the effector proteins that is transferred into host plant cells during *Agrobacterium* infection [43]. Early results showed that VirD5 did not seem to be important for tumor formation [44,45]. However, recent reports strongly support that VirD5 has specific functions in plant cells [33,36]. VirD5 interferes with mitosis by localizing to the centromeres/kinetochores in the nucleus of the host cells through its interaction with the conserved protein Spt4, and promoting chromosomal instability as seen by the high-frequent loss of a mini-chromosome in yeast [34]. Concurrently, the latest reports showed that VirD5 stimulates the activity of the conserved mitotic Aurora kinase Ipl1 via interaction with it. The aurora kinase activity is known to cause spindle instability, explaining enhanced chromosome mis-segregation seen when VirD5 is present [35]. Based on our previous reports that VirD5 protects the T-complex from rapid degradation by competitive interaction with the transcription factor VIP1 [33], and prohibits recruitment of cap-binding proteins (CBPs) by competitive interaction with the transcription factor VIP2, we concluded that VirD5 affects plant gene expression to response to *Agrobacterium* infection as a transcriptional activator-like protein and through interaction with certain plant transcriptional regulators [33,37].

Our findings [33,37] prompted us to investigate which genes in plant cells may be regulated by VirD5. Through the sequence analysis of Arabidopsis genes with at least two D5REs on their promoters (500 bp upstream of the translational start site), we found several candidate host genes that were putatively regulated by VirD5 and involved in the *Agrobacterium* infection process (Supplementary Table S1). To verify our hypothesis, we tested the effect of the *Agrobacterium* virulence protein VirD5 on the expression of the above putative genes. We found that VirD5 induced the expression of *D5RF* (Arabidopsis *At3g49480* gene, Figure 1). The activation activity of VirD5 to the *D5RF.1* promoter was demonstrated by both yeast one-hybrid assays and dual-luciferase assays (Figure 4). Therefore, VirD5 was further confirmed as the transcription activator and the *Arabidopsis thaliana D5RF.1* was the first identified as the target gene of VirD5 in regulation. *D5RF.1* is a F-Box protein with six leucine-rich repeats and a FBD domain. We found that although the promoter region of *WIT2* (At1g68910) also contains two D5RE sequences, the expression of *WIT2* responded to *Agrobacterium* infection, but not to VirD5 (Figure 1). WIT2 is involved in the nucleocytoplasmic transport of Arabidopsis root tip cells [46], and the upregulation of the gene may be beneficial to the infection of *Agrobacterium tumefaciens*. Therefore, the effect of the gene *WIT2* on the infection process of *Agrobacterium tumefaciens* is also worthy of attention. Our unpublished results showed that WIT2 interacts with VirE3 in vivo and in vitro.

Alternative splicing may produce a great diversity of proteins to meet the requirements of plant development and stress environment [47]. In the annotation version TAIR10 of the *A. thaliana* genome, 5885 alternatively spliced genes were reported (http://www.arabidopsis.org/) [48]. Through bioinformatics analysis, we found that *D5RF* has two transcripts, renamed as *D5RF.1* and *D5RF.2*, respectively. We successfully cloned the two transcripts using RT-PCR with gene specific primers. The two deduced proteins possess the same C-termini (from 140–449 aa) and D5RF.2 has a N-terminal extension of 140 amino acids compared to D5RF.1. Both D5RF.1 and D5RF.2 had a typical F-Box structure (Figure 2). In addition, D5RF.1 and D5RF.2 were both localized in the nucleus (Figure 3). However, no D5RE was found at 2000 bp upstream of the translation start site of *D5RF.2.* Further studies are required for *D5RF.2*.

Ubiquitin proteasome system (UPS) controls a large number of biological processes in eukaryotic cells. The SCF (SKP1-CUL1-F-box protein) ubiquitin ligase complex is a key player in plant and pathogen interactions [8,13]. As a component of SCF ubiquitin ligase complex, F-box protein mediates the polyubiquitination of target protein and then degrades proteasome dependent proteins in eukaryotic cells [36]. VirF protein was found to be the first prokaryotic protein with an F-box in *Agrobacterium tumefaciens*, and it interacts with plant homologs of the yeast Skp1 protein, indicating that VirF functions in the SCF ubiquitin ligase complex [24,28]. VirF targets at least VirE2 and its associated VIP1 for proteasomal degradation [24]. Although the F-box protein plays an important role, up to now, only a few F-box proteins encoded by plant pathogens have been studied in depth. Understanding the role of the F-box effector in the corresponding pathogen infection process is very helpful to explain the molecular arms race between host plants and pathogens [49]. Notably, plants encode an unusually large number of F-box proteins. The model plant *Arabidopsis thaliana* possesses almost 700 F-box genes, which represent almost 2.3% of the protein-coding genes [50,51]. A large number of studies have shown that UPS may regulate the host defense response by controlling the stability of host or pathogen proteins. In addition, increasing evidence suggests that several plant pathogens use host UPS for effective infection, which further emphasizes the importance of UPS in plant pathogen interaction [36]. Consistent with this concept, host UPS plays a critical role in *Agrobacterium* tumefaciens and plant interaction. Some studies have shown that host plants can upregulate or downregulate several UPS associated genes [52,53], some of which probably affect the efficiency of the *A. tumefaciens* infection [25,52]. Therefore, *A. tumefaciens* represents a powerful model system, which can be used to study how plants use UPS to prevent invasive pathogens and how pathogens exploit the host UPS in the process of infection [49]. For the D5RF as a typical ubiquitinated E3 ligase, more experiments are needed to elaborate its function as an enzyme. We tried to find the interacting proteins or substrates of D5RF by yeast two hybrid systems. Unfortunately, we did not succeed. Further studies are needed on the specific substrate of D5RF.

As VirD5 is one of those proteins exported from *Agrobacterium* to plant cells during the process of *Agrobacterium* infection, and affects the stable but not transient transformation efficiency of *Agrobacterium* [33,36], whereas D5RF.1 is the downstream target of VirD5 protein, we want to know whether D5RF will also participate in the process of *Agrobacterium* infection. Our results showed that, similar to VirD5, D5RF also affects the stable but not transient transformation efficiency of *Agrobacterium* (Figure 5 and Figure 6). D5RF is a protein that has never been studied. Its function in plant growth and development is completely unknown. In this report, we performed a transcriptome analysis using whole Arabidopsis seedlings of both *d5rf* and wild type Columbia. Using RNA-Seq to compare mutants and wild types, we identified numerous DEGs (Differentially Expressed Genes). By using GO enrichment analysis, we determined that the D5RF was involved in plant growth and developmental processes such as cell-wall biogenesis and metabolic process. In the process of evolution, plants have developed many elaborate defense strategies. However, as a successful pathogen, *Agrobacterium* has also evolved many ways to promote its infection. The interaction between plant and *Agrobacterium* is delicate and complicated. Further experiments are still required to elucidate the detailed functions of VirD5 and its associated plant proteins during *Agrobacterium* infection. Our data herein proved experimentally that VirD5 can act as a transcription factor to bind to the promoter region of *D5RF.1* and regulate its expression, proving that D5RF plays an important role in the process of *Agrobacterium* infection, and provided new insights into the strategy of *Agrobacterium* infection.

## 4. Materials and Methods

### 4.1. Plant Materials and Growth Conditions

All *Arabidopsis thaliana* lines used in this study were in the Columbia ecotype. The mutants of *d5rf* (CS24857, renamed as *d5rf*) was obtained from SALK (ABRC). Arabidopsis materials were grown under long photoperiod conditions (16 h light/8 h dark) or short photoperiod conditions (8 h light/16 h dark) at 22 °C. Seedlings were grown on Murashige and Skoog plates containing 3% sucrose and 0.8% agar or pots containing mixture of vermiculite and peat soil (1:1 by volume). All primers used in this study were listed in Supplementary Table S2.

### 4.2. Yeast One-Hybrid Assay

In yeast one-hybrid assay, the PGADT7:VirD5 and pAbAi reporter under the control of *D5RF.1* promoter (Supplementary Table S2) were co-transferred into yeast strain Y1H Gold. The transformed yeast cells were first spotted on SD minus Uracil and Leucine (SD/-Ura-Leu) medium, and then the cultured yeast colonies were resuspended in sterile ddH_2_O, adjusted to an OD_600_ of 0.8, and transferred onto SD/-Leu-Ura minimal medium and SD/-Leu-Ura minimal medium with 500 ng.mL^−1^ Aureobasidin A for culture. Qualitative colony-lift filter assays was performed according to the manufacturer’s protocols (Clontech, Protocol No. PT3024-1).

### 4.3. Dual-Luciferase Reporter Assay System

Transient expression for quantification of promoter activity in plants was performed as described [54]. The reporter vector was constructed in pGreenII 0800-LUC by inserting the *D5RF.1* promoter to the upstream of the LUC fragment. The effector vector was constructed in pGADT7 vector by inserting the VirD5 CDS to the downstream of the 35S promoter. The empty pGADT7 vector was used as the control. The reporter and effector vectors were used to transform *Agrobacterium* GV3101 separately. Both reporter and effector transformed *Agrobacterium* GV3101 were cultured on YEP medium with 50 μg mL^−1^ kanamycin and incubated at 28 °C for two days. Before infiltration, 100 μL of each culture (OD_600_ = 0.8) was mixed and a 20 μL loop of confluent *Agrobacteria* was re-suspended in 20 mL of infiltration media (10 mM MgCl_2_, 0.5 μM acetosyringone), cultured to an OD_600_ of 0.2, and incubated at room temperature without shaking for 2 h. *Nicotiana benthamiana* plants were grown under long photoperiod conditions (16 h light/8 h dark) at 22 °C until they had six leaves. The youngest leaves over 1 cm long were infiltrated with the *Agrobacterium* mixture and maintained in a glasshouse for the duration of the experiment. Approximately 300 μL of *Agrobacterium* mixture was permeated into young *N.benthamiana* leaves and transient expression was assayed from 3 to 14 days after inoculation.

### 4.4. Agrobacterium-Mediated Plant Stable and Transient Transformation Assay

Stable transformation assay: The *A. tumefaciens* tumorigenic strain A208 and A281 were used for tumorigenesis assays on wild-type (WT) Arabidopsis or Arabidopsis T-DNA insertion mutant *d5rf*. Arabidopsis root tumorigenesis assay was performed as described by Stanton B. Gelvin [55] with some modification. *Arabidopsis thaliana* was cultured in 1/2 Murashige and Skoog (MS) medium. After 10–14 days, the roots were cut into 3–5 mm segments. The root segments from five plants were pooled and infected for 15–20 min with A208 or A281 that was pre-adjusted to OD_600_ = 0.5 ± 0.05 with sterile ddH_2_O. After two days, the segments were arrayed on plates containing MS medium plus 100 mg L^−1^ Timentin or Carbenicillin, incubated at 28 °C (A208) or 22 °C (A281) for 3–4 weeks, and then scored for tumor development. The stable transformation efficiency was calculated as: no. of root segments with tumor/no. of root segments infected. All infection experiments were repeated at least three times. All of the data are means ± SD from three biological replicates. The data were made into graphs using GraphPad PRISM 5 software (La Jolla, CA, USA).

Transient transformation assay: Arabidopsis root transient transformation and β-glucuronidase (GUS) histochemical staining experiments were performed as described by Li et al. [56]. The Arabidopsis root segments were prepared as above for stable transformation assay. The root segments from five plants were pooled and infected for 15–20 min with EHA105 carrying *uidA* gene that was pre-adjusted to OD_600_ = 0.2 with sterile ddH_2_O. After co-cultivation on hormone-free MS medium for 2d or 7d, the root segments were transferred on MS containing 100 mg L^−1^ Timentin for 2 days, then stained with X-Gluc overnight at 37 °C. The root segments were washed with 70% ethanol and the blue-stained segments were counted using a stereomicroscope. The transient transformation efficiency was calculated as: no. of root segments stained in blue/no. of root segments infected. All infection experiments were repeated at least three times. The results were reported as percent positive ± standard error. The data were analyzed and made into graphs by GraphPad PRISM 5 software (La Jolla, CA, USA).

### 4.5. Subcellular Localization Assay in Arabidopsis Protoplasts

The vector pM999-YFP was used to study the subcellular localization of D5RF. The transient expression vector were constructed: 35S::D5RF.1-YFP and 35S::D5RF.2-YFP (Supplementary Table S2 lists the primers used for the vector construction). The empty vector (YFP, as control) and the above constructs were separately used to co-transform Arabidopsis protoplasts with 35S::Ghd7-CFP. The 35S::Ghd7-CFP was used as a nuclear localization marker. The methods for Arabidopsis protoplast isolation and transformation were as described by Yoo et al. [57] with modification. Arabidopsis plants were grown on soil in a greenhouse under a short photoperiod (8 h light/16 h dark at 22 °C). The well-expanded leaves (usually the fifth to seventh of true leaves) from 3–4-week-old plants (before flowering) were chosen. Leaf strips of 0.5–1-mm from the middle parts of leaves were digested in digestion solution (0.4 mol/L mannitol, 10 mmol/L 4-morpholineethanesulfonic acid (MES), 1.5% cellulose R-10, 0.75% macerozyme R-10, 0.1% BSA, and 1 mmol/L KCl_2,_, pH 5.7,) in the dark at room temperature in a desiccator under vacuum for 30 min and without vacuum for at least 3–4 h. The digestion sample was mixed with W5 solution (154 mmol/L NaCl, 125 mmol/L CaCl_2_, 5 mmol/L KCl, 2 mmol/L MES, pH 5.7), incubated at 25 °C, 80 rpm for 10 min, and filtered through a 300-mesh filter (50 μm). The protoplasts were collected by centrifugation at 100× *g* and 4 °C for 8 min. The supernatant was removed and the pellet was resuspended in another 5 mL W5 solution. The protoplasts were collected after another centrifugation at 100× *g* and 4 °C for 8 min, and resuspended in MMG solution (0.4 mol/L mannitol, 15 mmol/L MgCl_2_, 4 mmol/L MES, pH 5.7) to a final concentration of 2.0 × 10^5^/mL. For transformation, 5 μL of each endotoxin-free plasmid (5–10 μg) was pooled and gently mixed with 100 μL of protoplasts and 110 μL of PEG (Polyethylene Polyglycol)-CaCl_2_ solution (40% PEG4000, 0.4 mol/L mannitol, 100 mmol/L CaCl_2_), and then incubated at 25 °C in the dark for 15 min. The transformation mixture was mixed well with 400–440 μL W5 solution by gently rocking at room temperature to stop the reaction, and centrifugated at 100× *g* at room temperature for 2 min using a bench-top centrifuge. The supernatant was removed. The transformed protoplasts were re-suspended in WI solution (0.4 mol/L mannitol, 4 mmol/L KCl, 4 mmol/L MES, pH 5.7) and maintained in 6-well culture plates at 25 °C for 12–16 h in the dark. The subcellular localization analyses were performed with a confocal laser scanning microscope (Zeiss LSM data server). Fluorescence was excited at 458 nm (cyan fluorescent protein, CFP) and 514 nm (YFP), and emissions were detected from the following wavelength ranges: 465–480 nm (CFP) and 505–530 nm(YFP), as described previously [33]. For each subcellular localization analysis, at least three repeats were performed.

### 4.6. Reverse Transcription PCR and RNA-Sequencing

Arabidopsis plants were grown on soil in a greenhouse under a short photoperiod (8 h light/16 h dark at 22 °C). The rosette leaves of 45 day-old plants before flowering were used for RT-PCR. Infiltration was performed as described by Jones [58] with modification. *Agrobacterium* strains A281, A281-virD5, EHA105, and its *virD5* deletion mutant strain EHA105-*vird5* carrying a binary vector pCAMBIA1302 or pCAMBIA1302:VirD5 were activated by inoculating in YEP medium plus 50 mg. ML^−1^ of Rifampicin and culturing at 28 °C. After OD_600_ reached 1.0–1.2, the bacteria were collected by centrifugation at 2000× *g* for 5 min, resuspended to OD_600_ of 1.0 with 10 mmol/L MgCl_2_ solution, and kept at room temperature for 1 h. The 10 mmol/L MgCl_2_ solution was used as Mock. Each bacterial (or Mock) solution was used to infiltrate the back sides of the rosette leaves of eight Arabidopsis plants using 1 mL syringe without needle till the whole leaves filling with solution. After infiltration for 48 h, the leaves were collected, placed in a precooled ceramic mortar, ground into powder with liquid nitrogen. RNA samples were extracted using Trizol (Invitrogen, Waltham, MA, USA), according to the manufacturer’s instructions. cDNA synthesis was performed using 2 μg of RNAs and the Prime Script RT Reagent Kit with gDNA Eraser (Takara, Japan). CFX96 Real-Time System (Bio-Rad) and SYBR Green Realtime PCR Master Mix (TOYOBO, Shanghai) were used for real-time PCR analysis using the gene-specific primer sets indicated in Supplementary Table S2. Each assay was quantified in triplicate and normalized using *Actin* (*AT3G18780*) as an internal control. Three technical replicates were evaluated for each sample and the data are shown as the average ± SD of three biological replicates. The RT-qPCR profiles included the following steps: 94 °C for 3 min, followed by 45 cycles at 94 °C for 15 s, 60 °C for 15 s, and 72 °C for 15 s.

The methods for AGROBEST (an efficient Agrobacterium-mediated transient expression method for versatile gene function analyses in Arabidopsis seedlings) were as described by Wu et al. [59] with modification. Seedlings growing on half MS basal medium agar plates for 10 days were used for RNA sequencing. Transcriptomic sequencing was performed by lllumina HiSeq 2500 technique. RNA samples were submitted to the Novogene Company (Beijing, China), where library preparation and high-throughput sequencing services were performed. The *Arabidopsis thaliana* TAIR10 genome GFF3 annotation file (https://www.arabidopsis.org/download_files/Genes/TAIR10_genome_release/TAIR10_gff3/TAIR10_GFF3_genes.gff) was used as a reference. The gene expression levels were calculated using the reads per kilo bases per million reads (RPKM) method. EdgeR software was applied to identify the differentially expressed genes (DEGs) between the libraries. The fold change (|log2FC| ≥ 1) and p-value (*p* ≤ 0.05) were used as the indexes of statistical significance. Gene Ontology (GO) enrichment analysis of differentially expressed genes was implemented by the clusterProfiler R package, in which gene length bias was corrected. GO terms with corrected P-value less than 0.05 were considered significantly enriched by differential expressed genes. Supplementary Materials can be found at https://www.ncbi.nlm.nih.gov/geo/query/acc.cgi?acc=GSE155112. Supplementary Table S2 align_pct and align_ region; Supplementary Table S3 *d5rf* vs. col_0_deg_all; Supplementary Table S4 *d5rf* vs. col_0_Goenrich; and Supplementary Table S5 *d5rf* vs. col_0_KEGGenrich. The RNA-Seq raw data were deposited in NCBI’s Sequence Read Archive (SRA) with accession code PRJNA647639.

## Supplementary Materials

Supplementary materials can be found at https://www.mdpi.com/1422-0067/21/18/6731/s1.

## Abbreviations

ABA	Abscisic Acid
DEGs	Differentially Expressed Genes
D5RE	VirD5 response element
D5RF	VirD5 response F-box protein
RT-PCR	Reverse Transcription-Polymerase Chain Reaction
RT-QPCR	Real-time quantitative PCR
WT	Wild Type
YFP	Yellow Fluorescent Protein
Y2H	Yeast Two-Hybrid
