# Supplementary material for: F-Box Gene D5RF Is Regulated by Agrobacterium Virulence Protein VirD5 and Essential for Agrobacterium-Mediated Plant Transformation
Source: Int J Mol Sci. 2020 Sep 14;21(18):6731. doi: 10.3390/ijms21186731 (PMC7555846; doi:10.3390/ijms21186731)
Supplement: Supplementary file 1 [file ijms-21-06731-s001.zip › Supp for conversion/Supp for conversion.pdf]

International Journal of *Molecular Sciences*

MDPI

F-box gene D5RF is regulated by Agrobacterium virulence protein VirD5 and essential for Agrobacterium-mediated plant transformation

Shaojuan Zhang, Zhuo Chen, Fei Huang, YaFei Wang and Meizhong Luo*

College of Life Science and Technology, Huazhong Agricultural University, Wuhan 430070, China;
*Author for correspondence: Meizhong Luo
Tel: +86 27 87284213 (office)
Email: mzluo@mail.hzau.edu.cn

Supporting information

Supplementary materials can be found at https://www.ncbi.nlm.nih.gov/geo/query/acc.cgi?acc=GSE155112.
Supplement Table 2 align_pct and align_ region;
Supplement Table 3 *d5rf* vs col_0_deg_all;
Supplement Table 4 *d5rf* vs col_0_Goenrich;
Supplement Table 5 *d5rf* vs col_0_KEGGenrich.
The RNA-Seq raw data are deposited in NCBI'S Sequence Read Archive (SRA) with accession code PRJNA647639.

Table S1. The putative genes directly regulated by VirD5.

Gene	Protein	No. of D5RE	Annotated functions
At2g04230	--	2	F-box and LRR protein
AT5G16690	*ATORC3*	2	Involved in double-strand break repair
At3g19210	*RAD54*	2	a member of the SWI2/SNF2 family of DNA-stimulated ATPases
At5g49160	*DDM2/MET1*	2	cytosine methyltransferase
At3g49480.1	*D5RF*	2	F-box/LRR-repeat protein
At1g68910	*WIT2*	2	WPP domain-interacting protein 2

Note: D5RE indicates VirD5 response element (CCGCNG).

Table S2. List of oligomucleotides.

Gene name	Primer name	Sequence	Restriction site
AT3G49480.1	p49480-F2	5'-GGggtaccTTACATAGAAACCACTAGTGACG-3'	*Kpn*I
	P49480-R2	5'-CCGctcgagTCTGATCGGCTCTCCTTTC-3'	*Xho*I
	49480-LUC-F3	5'-GGggtaccTACCGGATGCGATATGGCAG-3'	*Kpn*I
	49480-LUC-R3	5'-CGggatccTCTGATCGGCTCTCCTTTCCA-3'	*Bam*HI
	49480-F4	5'-GGggtaccATGCCTAAGAGCTTTTACTT-3'	*Kpn*I
	49480-R4	5'-GGggtaccATATCTAAATGACATTCGAGC-3'	*Kpn*I
	49480-QF1	5'-GTTGGAATCACTTTTGGTTTTGCTC-3'	-
	49480-QR1	5'-ACATCTTGGAATAGAACTCGGTTGA-3'	-
AT3G49480.2	L49480-F1	5'-GGggtaccATGACAGAACTCGTCGAACATAG-3'	*Kpn*I
	L49480-R1	5'-CATGccatggATATCTAAATGACATTCGAGCAG-3'	*Nco*I
AT5G16690	16690-QF1	5'-TGATAAGGTTGCCACAGTCATAGAA-3'	-
	16690-QR1	5'-GGTCTCCAAGTAAAGCAGATTGAAG-3'	-

AT5G49610	9160-QF1	5'-GAGTTTGCAGGGAACATAAATCACA-3'	-
	9160-QR1	5'-TTCTTGAGATGTAGGGCTTCTTTGA-3'	-
AT3G19210	19210-QF1	5'-ACATGGGTTTAGGGAAGACATTACA-3'	-
	19210-QR1	5'-TCCTGTCTCCAACCCATTTCTTAAT-3'	-
AT1G68910	8910-QF1	5'-GAAATGGAAAATGCAAATGAGTCGG-3'	-
	8910-QR1	5'-ACTTCTAATTCCCTGACTTGCTTCT-3'	-
AT2G04230	4230-QF1	5'-AGAGTTAGAGGAGGCAAAAATCAGT-3'	
	4230-QR1	5'-TTTAACCTCAAGGGGTGATAAGTGT-3'	
VirD5	D-F03	5'-ggatccATGACAGGAAAGTCGAAAGTT-3'	*Bam*HI
	D-R03	5'-gtcgacTCAGCGTTTAAACGCTTTGTC-3'	*Sal*I
AT1G70880	70880-QF	5'-GGCTCACCCTGAAACTCTCC-3'	
	70880-QR-	5'-ACGTGCGGCTCAGTGTTTAT-3'	
AT1G70890	70890-QF	5'-GGGAGTCAGGGAGTATTGCG-3'	
	70890-QR-	5'-CTCGGCCAAGAGATGTTCGT-3'	
AT2G01520	1520-QF	5'-ACACAAGGAAGAGAGATGGCG-3'	
	1520-QR	5'-GGGGAAGAGGTGGTTCTCAC-3'	
AT2G01530	1530-QF	5'-AAGAAGCGCACACACACAAC-3'	
	1530-QR	5'-GTCGTGGACAGTGACACCTT-3'	
AT2G02120	2120-QF	5'-TCAGCCGTTCTTTTCTTGGTG-3'	
	2120-QR	5'-GCTCACGCATTTACCCTTGA-3'	
AT3G26460	26460-QF	5'-CAACATCACCATCATCCCTATATGT-3'	
	26460-QR	5'-AGAAAGTAACTCCACAAGACCCA-3'	
AT5G36910	36910-QF	5'-GCATGCTTTCCGTGTCATCC-3'	
	36910-QR	5'-TTGGAGAGTGGTCAAGGCAC-3'	
AT5G44430	4430-QF	5'-TGGTGCTTGATCGTGTGTGTA-3'	
	4430-QR	5'-AACAGTCCAACGTCGTACATAA-3'	

```
-500 TTACATAGAAACCACTAGTGACGTAAAAAGCAATACTGTTTGGTGGTTTCTTGAAGAGTCATTCCGTTT
-431 TCACAAGGCACCTTTGTTAGAACGTTTGTGGATCGACCTCGGTCCACAATGTCCTATTAATGTTAATCC
-362 TGTAAAGTGGGTTGCAAAAGTACTTGATACCAGGGGCGGATCTAGAATGTAATTTAATGTGGGGCACCA
                                      VirD5 response element
-293 TATATATAAATAGTACTAAGTATAATTTAATTACCTAAATTGTATTGTGATTATAAATTATTTTTACAA
-224 AAATAACAGTGCGGAGACTTGAACTCGGGTTAGTATGAGGCACTTTTTGCTGGAAAACCATTTGAGTTA
        VirD5 response element
-155 CCTGTATTACATTGTTTAGTAGTAGCTTATAAAATATTTATAGAATTTAGTATCAGGCACGTGCCCCAC
 -86 CTGCCTCCAACGTGGGTCCGCCACTGCTTGATACTCGTGTGCGTTGGCTAACTTTCAGGCTCCTTTGGA
 -17 AAGGAGAGCCGATCAGA
```

Figure S1. The promoter sequence of *D5RF.1* gene (500 bp upstream of the translational start site), On the chromosome 3: 18342043-18342543. The red sequences are the possible binding sites of VirD5 protein (D5RE).

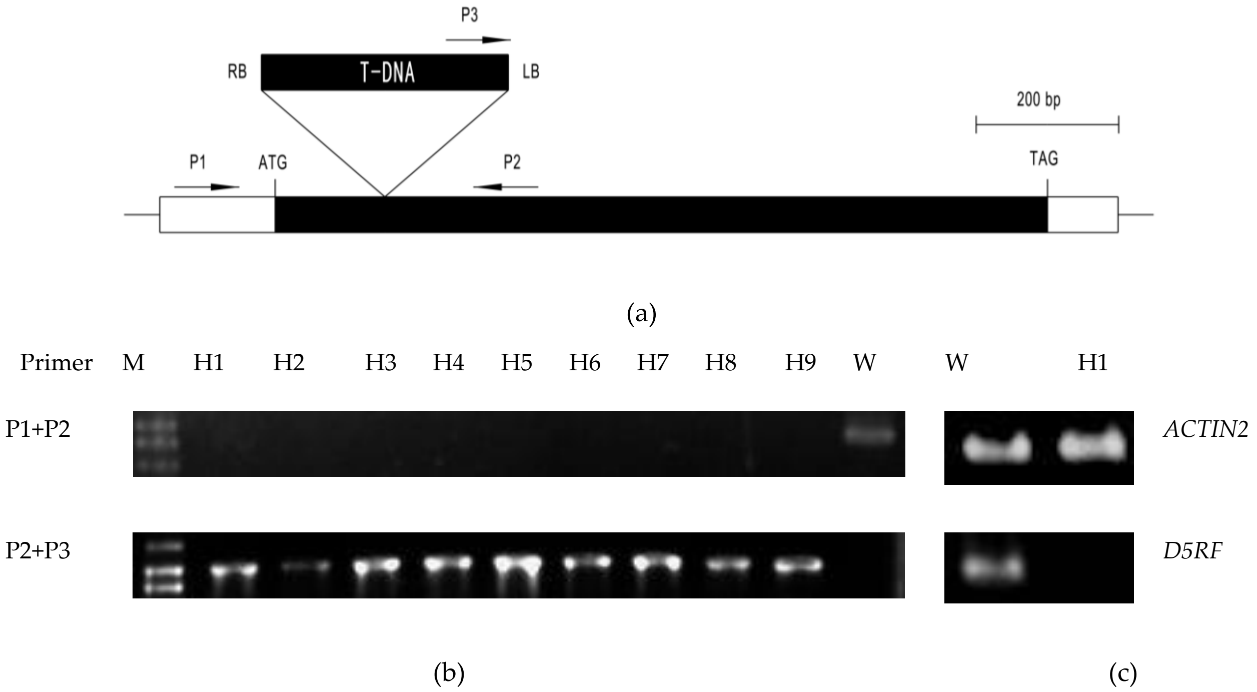

Figure S2. Identification of the *d5rf* mutant. (a) *D5RF.1* structure. The T-DNA was inserted into the exon approximately 200 bp downstream of the translational start site. P1, P2 and P3 indicate primers used for genotyping; RB and LB indicate the T-DNA right border and left border, respectively. (b) PCR genotyping of *d5rf* mutants. M indicates Marker. W and H indicate the wild-type and homozygous *d5rf* mutant plants, respectively. (c) RT–PCR analysis of *d5rf* and wild-type plants. RT-PCR showed that the expression level of *D5RF* was depressed in the *d5rf* mutant. Actin was used as the control; W, wild-type; H, homozygous *d5rf* mutant.

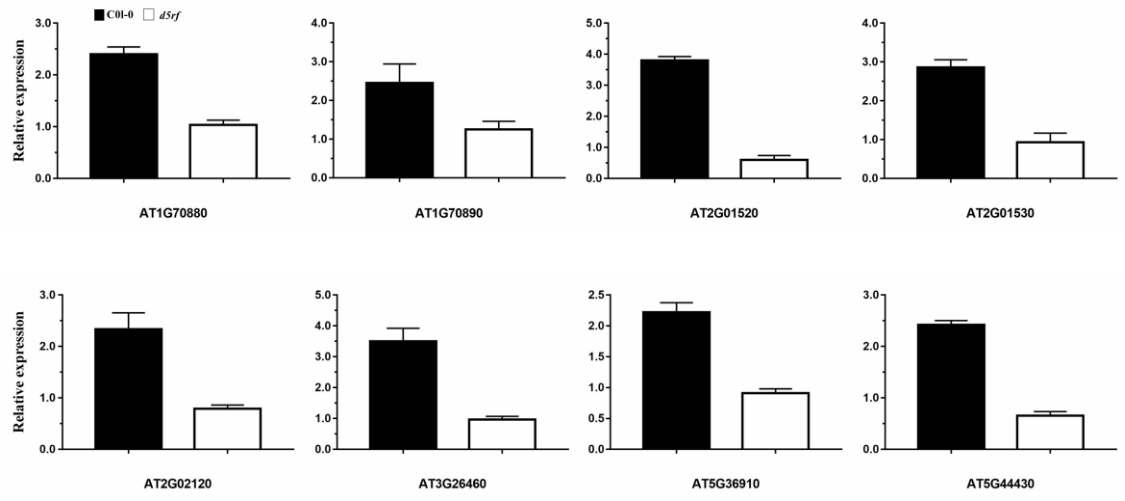

Figure S3. Validation of the RNA-Seq Data by Real-Time qRT-PCR. Eight different pathogen-responsive Genes genes that had less transcript abundance in *d5rf* compared with Col-0, based on RNA-Seq experiments, were selected for validation. Total RNA was extracted from leaves of wild-type Col-0 and *d5rf*. The first-strand cDNA was synthesized and used for qRT-PCR using gene-specific primers. The data represent the average of three biological replicates. each biological replicate includes three technical replicates. SE values were shown as error bars.
